# Respiratory syncytial virus A genotype classification based on systematic intergenotypic and intragenotypic sequence analysis

**DOI:** 10.1038/s41598-019-56552-2

**Published:** 2019-12-27

**Authors:** Juan Carlos Muñoz-Escalante, Andreu Comas-García, Sofía Bernal-Silva, Carla Daniela Robles-Espinoza, Guillermo Gómez-Leal, Daniel E. Noyola

**Affiliations:** 1 Microbiology Department, Facultad de Medicina, Universidad Autónoma de San Luis Potosí, San Luis Potosí, Mexico; 2 Center for Research in Biomedicine and Health Sciences, Facultad de Medicina, Universidad Autónoma de San Luis Potosí, San Luis Potosí, Mexico; 3 International Laboratory for Human Genome Research, Universidad Nacional Autónoma de México, Querétaro, Mexico

**Keywords:** Viral infection, Viral epidemiology

## Abstract

Respiratory syncytial virus (RSV), a leading cause of lower respiratory tract infections, is classified in two major groups (A and B) with multiple genotypes within them. Continuous changes in spatiotemporal distribution of RSV genotypes have been recorded since the identification of this virus. However, there are no established criteria for genotype definition, which affects the understanding of viral evolution, immunity, and development of vaccines. We conducted a phylogenetic analysis of 4,353 RSV-A G gene ectodomain sequences, and used 1,103 complete genome sequences to analyze the totallity of RSV-A genes. Intra- and intergenotype p-distance analysis and identification of molecular markers associated to specific genotypes were performed. Our results indicate that previously reported genotypes can be classified into nine distinct genotypes: GA1-GA7, SAA1, and NA1. We propose the analysis of the G gene ectodomain with a wide set of reference sequences of all genotypes for an accurate genotype identification.

## Introduction

Respiratory syncytial virus (RSV) is the most common cause of lower respiratory tract infections (LRTI) in young infants, and a leading etiology for severe respiratory infections in children and older adults^1^. RSV was first described in 1956 and, since then, has been extensively studied as a cause of acute respiratory infections. RSV strains were initially classified in two groups (A and B) based on reactivity to monoclonal antibodies^2^. Subsequently, sequencing of viral genes allowed for more detailed characterization and classification of viruses^3–6^. As a result, multiple genotypes have been described within both RSV-A and RSV-B groups^7–10^. However, there is not an established system or a consensus to define RSV genotypes^11–13^. Of relevance, RSV has shown a dynamic process of evolution with notable changes through time with the appearance of new genotypes and apparent extinction of others. The most notable change observed in recent years is the emergence of RSV-A strains with a partial duplication of the G protein gene^14^. Although RSV-B strains with an analogous duplication have circulated for more than 20 years, the mechanisms leading to emergence of these viruses, as well as the epidemiological and clinical implications of the observed changes have not been entirely defined.

The G gene contains the genomic region that shows more diversity between viral strains, and is usually used to distinguish and classify RSV^15^. It has been postulated that RSV diversity is generated as a result of immune selection by antibodies directed against viral surface proteins, such as the G protein^4,16,17^. However, recent studies suggest that RSV variability may not be the result of such immune mechanisms^11^. RSV diversity is an important factor that allows for reinfections to occur throughout life and also has implications for design of diagnostic assays, antiviral therapies, and preventive strategies (passive immunization and vaccines). In order to analyze this, a clear understanding of the genetic diversity of available viral sequences and their correct genotype assignment should be of help. The need for a thorough analysis is highlighted by the lack of consensus regarding existing genotypes. Several authors have described the identification of new viral genotypes, but criteria used to define them are not uniform and have not been clearly established. Also, because the G gene shows the highest variability, allowing for strain classification, not so much attention has been dedicated to other viral genes. However, other genes also show substantial variation and changes in them could also be of help in genotype classification. In addition, variations in genes other than the G gene may contribute to strain virulence and to epidemiological circulation patterns through time^11,18^.

In recent years, the number of RSV sequences that are available in GenBank has increased importantly, including sequences of the G gene as well as of the complete viral genome. This allows to carry out more detailed analyses improving the accuracy of genotype classification. In this study, we analyzed all available RSV-A sequences that include the complete G gene ectodomain with the aim of defining the characteristics of previously described genotypes. In addition, sequences of the NS1, NS2, N, P, M, SH, F, M2, and L genes were analyzed and genetic markers associated to specific genotypes were identified. These results will help to establish a framework for RSV classification and allow for a better understanding of the mechanisms leading to the generation of diversity of this virus.

## Results

### Dataset

We analyzed all RSV sequences available in GenBank that included at least the RSV G gene ectodomain length, and excluded those which belonged to mutant, synthetic, chimeric, patented, or vaccine sequences, as well as those that corresponded to organisms other than RSV. Then we performed a local BLAST to identify those sequences that corresponded to the RSV-A G gene ectodomain. This resulted in 10,340 RSV downloaded sequences and 5,410 sequences that corresponded to RSV-A genotype which contained at least the G gene ectodomain. Analysis of these sequences showed that 996 of them contained gaps or degenerate nucleotides within the G gene ectodomain region; therefore, they were excluded from further analysis resulting in a final dataset of 4,414 sequences useful for genotype assignment. In addition, for 1,103 of these G gene sequences, the complete viral genome was available (corresponding to 24.98% of the dataset) and was used to analyze each individual gene including complete gene cladogram inference, p-distance matrix computation, and identification of molecular markers (from nucleotides and amino acids sequences). The number of unique and duplicated sequences of each gene included in the dataset is shown in Supplementary Fig. 1.

### Genotype assignment

**Figure 1:**
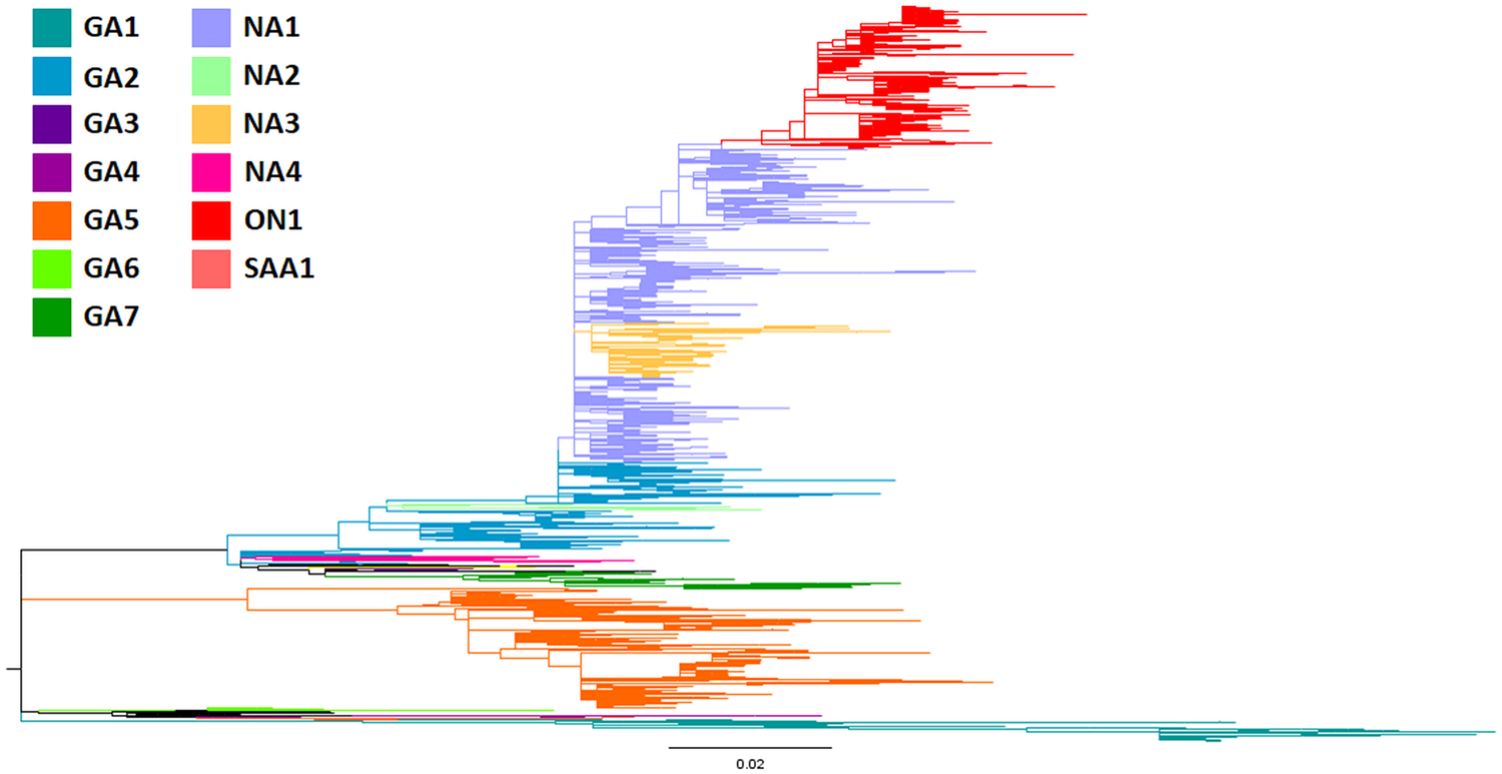
Phylogenetic analysis of RSV group A. Maximum Likelihood Cladogram of 2,151 unique nucleotide sequences of the G gene ectodomain retrieved from GenBank. Clades are colored according to the genotype assignment.

**Figure 2:**
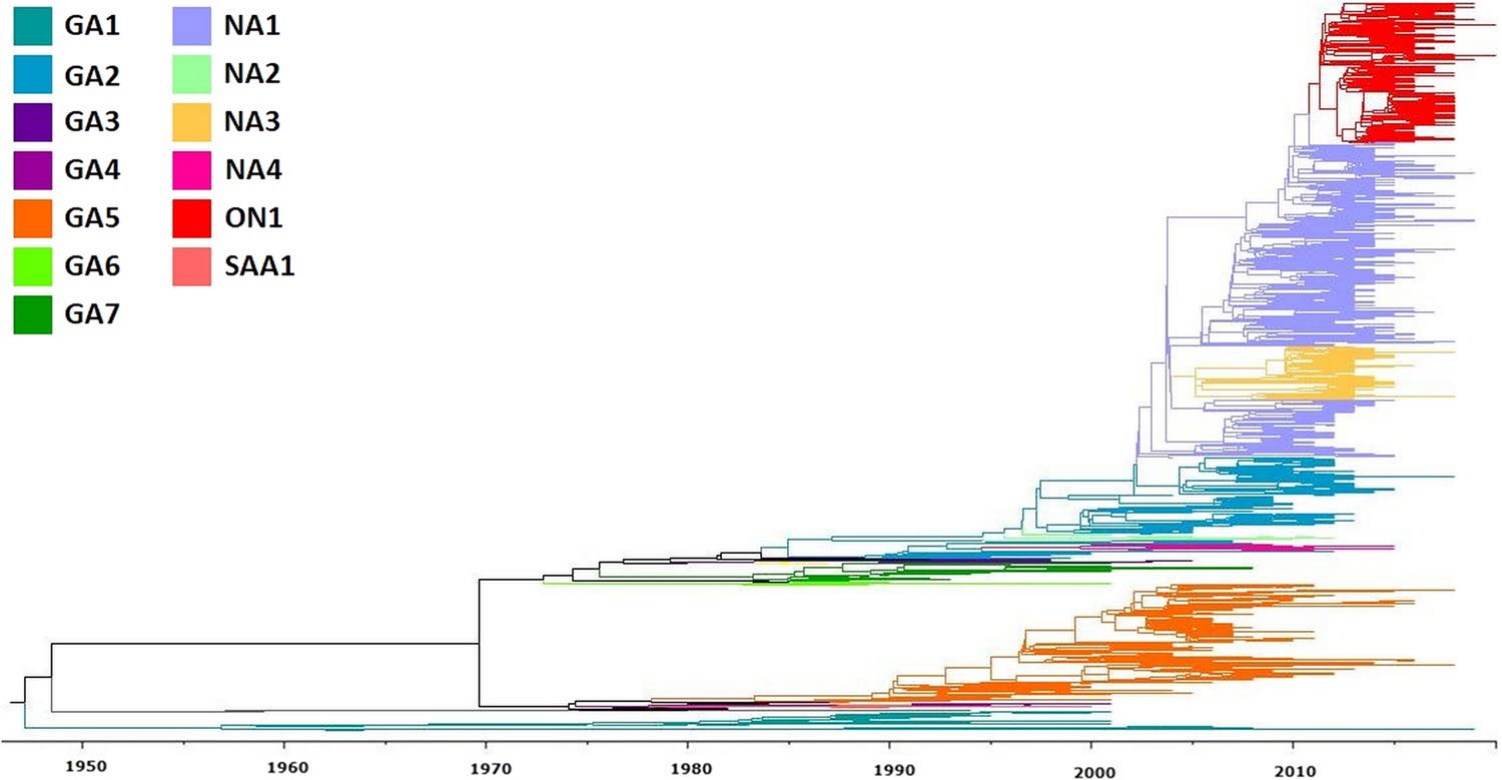
Phylogenetic analysis of RSV group A. Maximum Clade Credibility (MCC) Tree from Bayesian analysis of 2,151 unique nucleotide sequences of the G gene ectodomain retrieved from GenBank. Clades are colored according to the genotype assignment.

The 2,151 unique G gene ectodomain sequences were analyzed and genotype assignment was carried out by sequence clustering with reference sequences in same clade. Analysis was carried out analyzing the topology of both Maximum Likelihood Cladogram and Maximum Clade Credibility Tree, and both assignment methods were concordant. The more abundantly represented genotype was NA1 with 35.5% of the total data set, followed by ON1 and GA5 that corresponded to 21.5% and 19% of the analyzed sequences, respectively. There were 61 sequences (2.8%) that did not cluster clearly with the selected 73 reference strains. The Maximum Likelihood Cladogram and Maximum Clade Credibility Tree from the Bayesian analysis are shown in Figs. 1 and 2.

**Figure 3:**
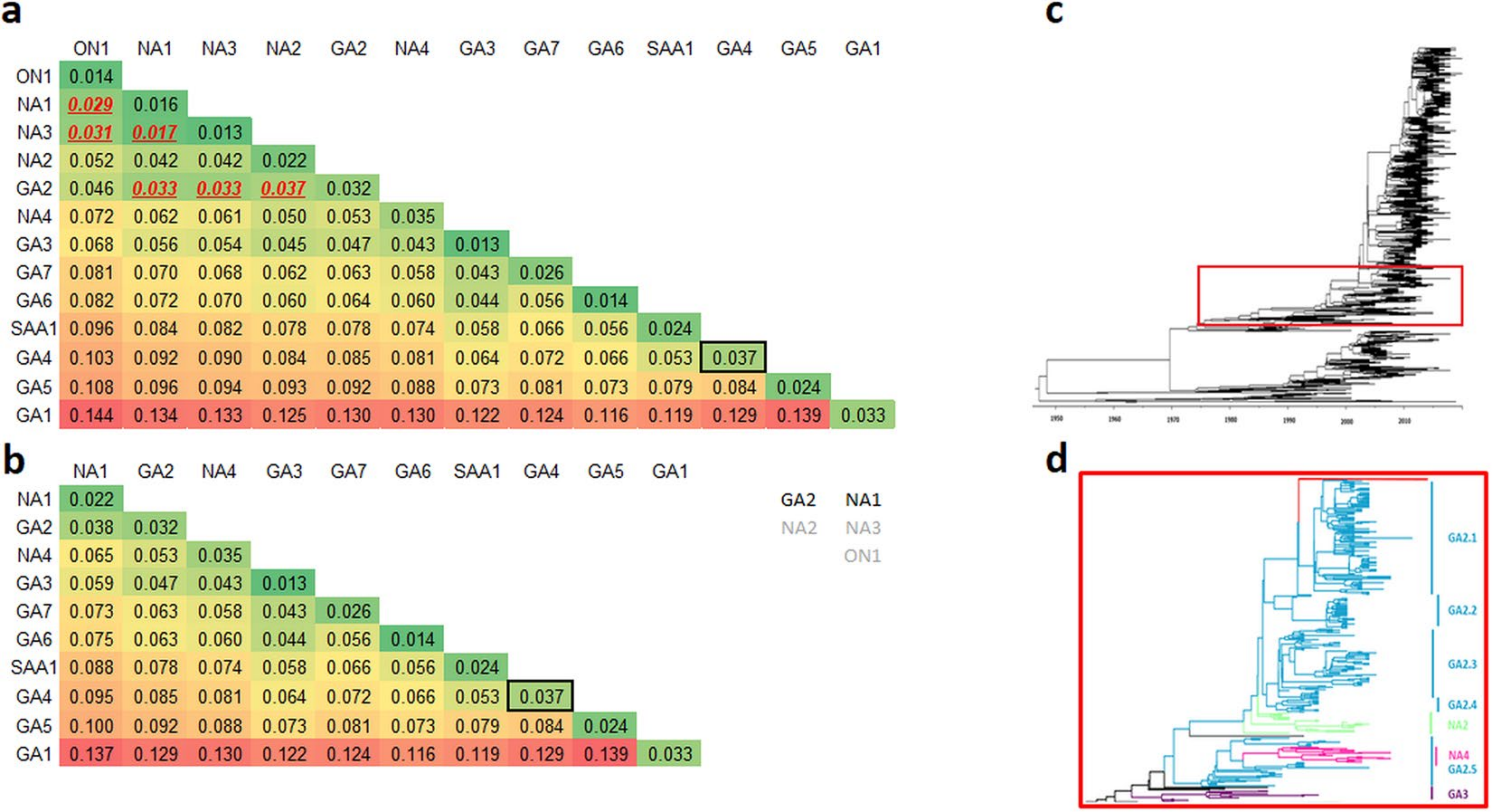
Proportional (p) nucleotide distances for the G gene calculated between and within RSV-A genotypes. Red font underlined p*-*distances indicates values below intragenotypic threshold distance (0.037) used for clustering sequences in the same genotype. (**a**) RSV-A distinct genotypes established using a stepwise closest neighbor joining strategy; the NA2 genotype was regrouped within GA2 genotype, and the NA3 and ON1 genotypes were regrouped within NA1 genotype. (**b**) RSV-A G gene ectodomain Maximum Clade Credibility (MCC) Tree showed that the GA2 genotype includes five distinct subclades (3c and 3d).

A p-distance matrix including intragenotype and intergenotype distance was computed for the genotype assigned dataset (N = 4,353). The largest intragenotype distance among the 13 recognized genotypes (GA1-GA7, NA1-NA4, ON1, SAA1)^6–8,14,19,20^ was obtained for GA4 (p-distance = 0.037) (Fig. 3a); therefore, this value was used as a threshold to identify those genotypes that belong to the same genotype. ON1, NA1, and NA3 showed p-distances lower than the threshold between each of them, indicating that they could be considered as part of the same genotype. GA2 also showed a lower p-distance compared to NA1 and NA3, but not compared to ON1. In addition, NA2 and GA2 showed a p-distance lower than the threshold; however, the p-distance between NA2 and NA1, NA3, and ON1 was higher than the threshold. In order to take this into account, we grouped sequences within the same genotype using a stepwise closest neighbor joining strategy beginning with the two genotypes with the lowest p-distance (NA1 and NA3; p-distance = 0.017) and repeating the analysis until all intergenotype distances were higher than the intragenotype threshold. This resulted in joining of genotypes GA2 and NA2, and of genotypes NA1, NA3, and ON1 (Fig. 3a,b).

Of note, analysis of the Maximum Clade Credibility tree and the Maximum Likelihood cladogram showed that sequences assigned as GA2 genotype (based on clustering with the reference sequences) did not constitute a unique clade, but a five subclade series originating from a common node with GA3 genotype (Fig. 3c,d).

**Figure 4:**
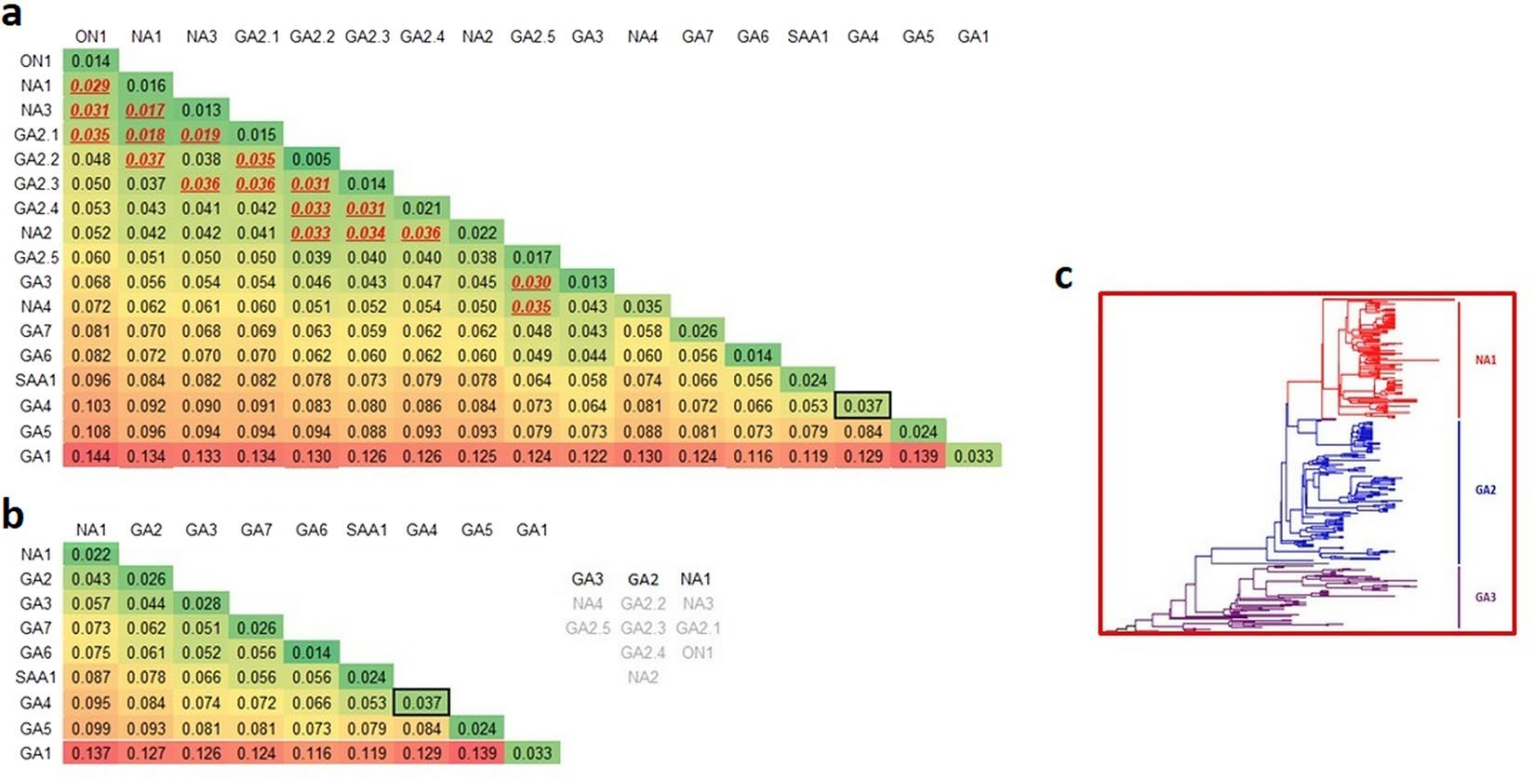
Proportional (p) nucleotide distances for the G gene calculated between and within RSV-A genotypes including GA2.1-GA2.5 clades separately. Red font underlined p*-*distances indicates values below intragenotypic threshold distance (0.037) used for clustering sequences in the same genotype. (**a**) RSV-A distinct genotypes established using a stepwise closest neighbor joining strategy; the NA4 genotype and GA2.5 subclade were regrouped with the GA3 genotype; the NA2 genotype and GA2.2, GA2.3, and GA2.4 were grouped together and assigned as the GA2 genotype; the NA3 genotype, GA2.1 subclade, and ON1 genotype grouped together with the NA1 genotype. (**b**) RSV-A G gene ectodomain Maximum Clade Credibility (MCC) Tree showing the clades that conform the new assignment of GA3, GA2, and NA1 genotypes (**c**).

Therefore, to elucidate if sequences that have been assigned as GA2 genotype are correctly assigned as a single independent genotype, a p-distance analysis that took into account these 5 subclades (identified as GA2.1-GA2.5) was carried out. The resulting p-distance matrix is shown in Fig. 4a. The stepwise closest neighbor joining strategy was used to regroup the GA2 subclades among them and with other RSV-A genotypes. This resulted in the joining of genotype NA2 and clades GA2.2, GA2.3, and GA2.4; genotypes GA3, NA4, and clade GA2.5; and genotypes NA1, NA3, ON1, and clade GA2.1 (Fig. 4b,c).

**Figure 5:**
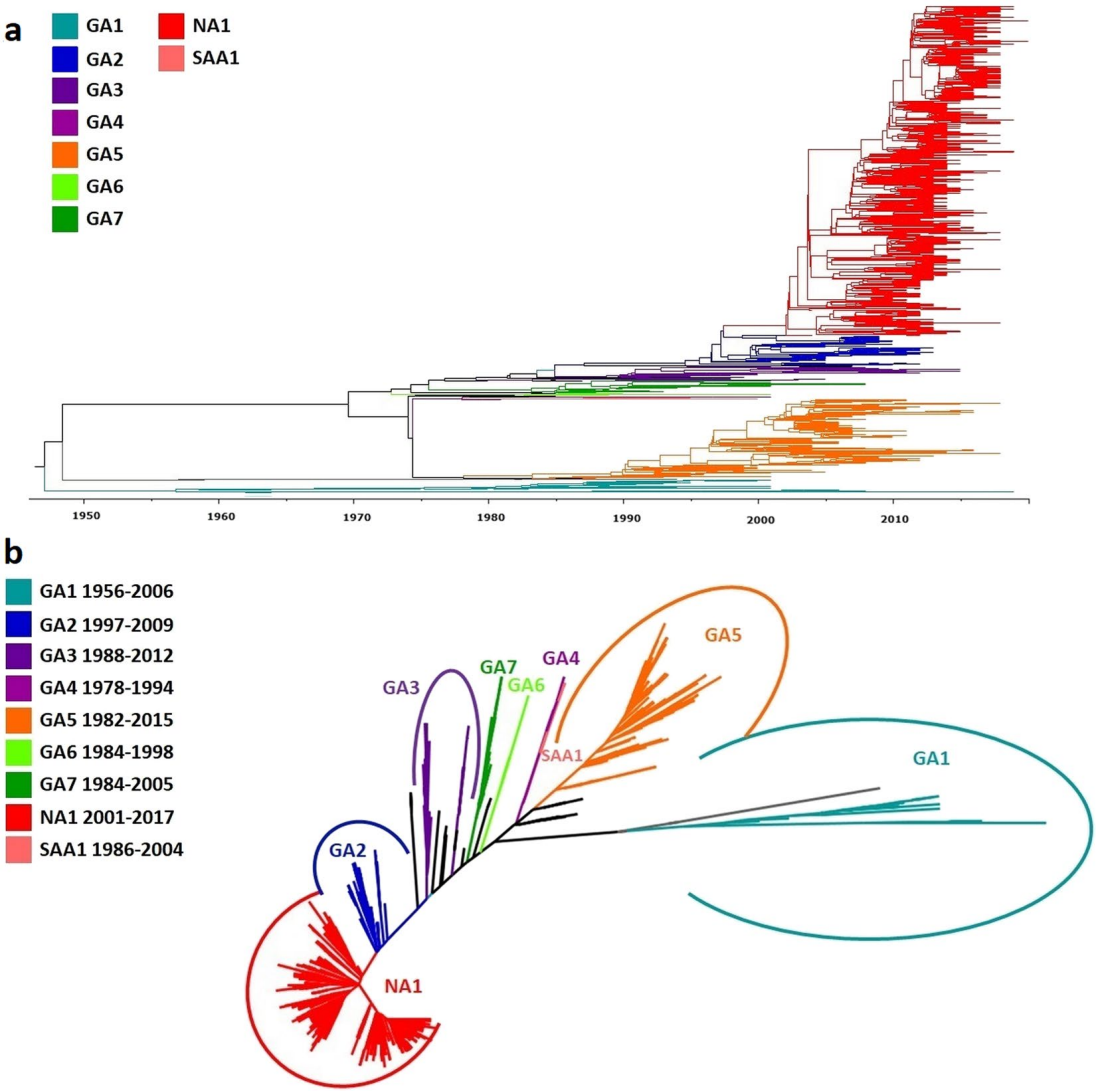
Phylogenetic analysis of RSV group A. Maximum Clade Credibility (MCC) Tree from Bayesian analysis of 2,151 unique nucleotide sequences of the G gene ectodomain retrieved from GenBank. Clades defined after genotype reassignment, based on p-distance grouping are shown in (**a**). Period of time (years) of circulation of each genotype based on the date of collection of samples from which the sequences were derived (**b**).

Based on the subclades and genotypes grouping, the new assignment resulted in the fusion of GA3, NA4, and GA2.5 named as **GA3**; NA2, GA2.2, GA2.3, and GA2.4 named as **GA2**; and NA1, NA3, ON1, and GA2.1 named as **NA1** (Fig. 5a). The unrooted phylogenetic tree in a radial representation of the genotypes with the new assignment, as well as the time span of circulation of each genotype based on the sample date from which the sequences were obtained is shown in Fig. 5b.

### Individual gene analysis

Individual gene cladograms were generated from the unique sequences out of 1,103 RSV-A complete genome sequences dataset using Maximum Likelihood method under the best suitable model for each gene data. For SH, G, F, M2 and L genes we constructed a Maximum Likelihood Cladogram using MEGA-X under a general time-reversible (GTR) substitution model and a discrete gamma distribution to model rate variation among sites. For P and M we used the Tamura Nei 1993 substitution model and a discrete gamma distribution to model rate variation among sites. For NS1 and N we used the Tamura 1992 substitution model and a discrete gamma distribution. For NS2 we used the Hasegawa, Kishino and Yano 1985 substitution model and a discrete gamma distribution. For each cladogram the reliability was determined through a bootstrap resampling analysis using 1,000 iterations. Due to the large size of L gene, sites conserved at 100% among all the sequences were removed from the alignment, and the cladogram for this gene was generated with the use of only polymorphic sites. In addition, p-distance matrices were generated for each gene; to carry out this analysis, sequences were assigned to genotypes based on the results described for the G gene ectodomain sequence of each viral isolate.

**Figure 6:**
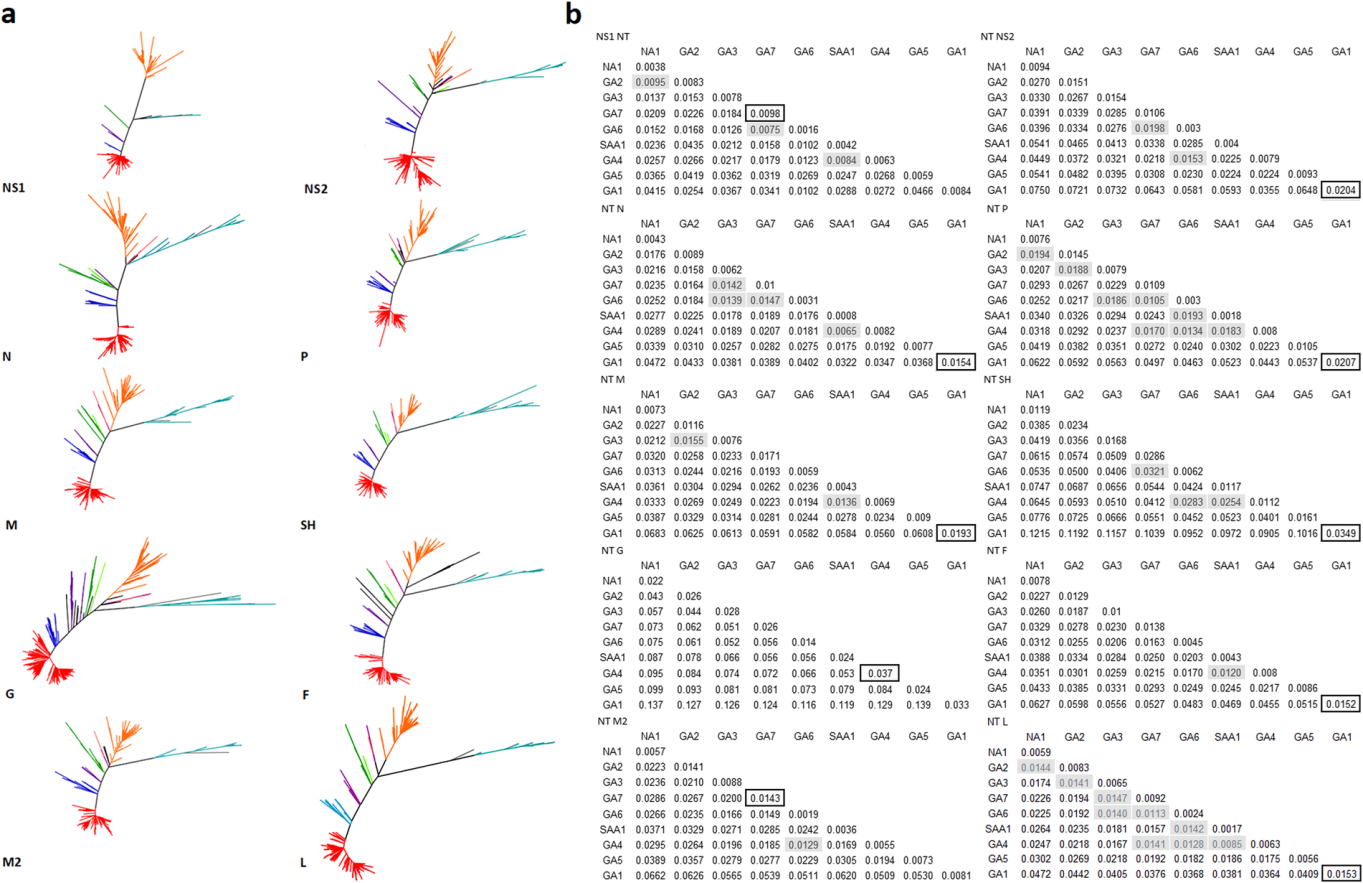
Phylogenetic analysis of RSV group A. Maximum Likelihood Trees for NS1, NS2, N, P, M, SH, G, F, and M2 genes. (**a**) Proportional (p) nucleotide distances calculated between and within RSV-A genotypes for each gene. (**b**) Values in black squares indicate the lowest intragenotype distance for each gene; gray shaded values indicate intergenotype p-distances that are lower than the intragenotype threshold established for each gene.

Topologies obtained in the cladograms of all individual genes were highly similar to each other and to the G gene ectodomain trees (Fig. 6a). Intragenotype and intergenotype p-distances were lower than those found in the analysis of the G gene ectodomain. This is consistent with the higher variability of the G gene compared to all other genes. Overall, the results obtained were consistent with genotype assignment based on the G gene analysis; in Fig. 6b, the higher intragenotypic p-distance is boxed for individual gene comparisons. Intergenotypic p-distances between GA1 and GA5, and all other genotypes were higher than the threshold established for each gene. Some discrepancies were observed among other genotypes in which intergenotypic p-distances were lower than the threshold in comparison to other genotypes in one or more genes (shadowed in Fig. 6b). Exceptions included genotypes GA4 and SAA1 which showed p-distance values between them lower than the intragenotype threshold in seven of the ten genes (NS1, N, P, M, SH, F, and L genes), genotypes GA6 and GA7 which showed values between them lower than the intragenotype threshold in six genes (NS1, NS2, N, P, SH, and L genes), and GA4 and GA6 which showed values lower than the intragenotype threshold in five genes (NS2, SH, M2, P, and L). Of note, comparisons in which the intergenotypic p-distance was lower than the threshold were registered more frequently for combinations of genotypes for which the number of sequences was small; this occurred in 16 (25%) of 64 comparisons in which there were <20 sequences available, compared to 17 (5.7%) of 296 comparisons with an n > 20 (P < 0.001). Also, values below the threshold were observed more commonly in genes with low evolutionary rates (such as the L gene) compared to those with high evolutionary rates (such as the G gene).

### Identification of molecular markers

In order to identify the presence of molecular markers, sequences of the ten complete genes, including UTRs, and their respective amino acid sequences were analyzed. Sequences were grouped by their previously assigned genotype, and every change at every site in comparison to the prototype Long strain sequence (ATCC VR-26) was registered. Subsequently, the fixation percentage of changes in every position for each genotype was calculated. If fixation percentage exceeded 75%, it was considered a molecular marker. Although a higher cutoff value (such as 90% or 95%) would be better suited to identify specific markers, we chose a cutoff value of 75% because the number of available sequences for some genotypes (GA4, GA6, SAA1) was small. To allow for better assessment of these results, information regarding the sensitivity and specificity for the identified amino acid molecular markers is provided (Supplementary Table 1).

**Figure 7:**
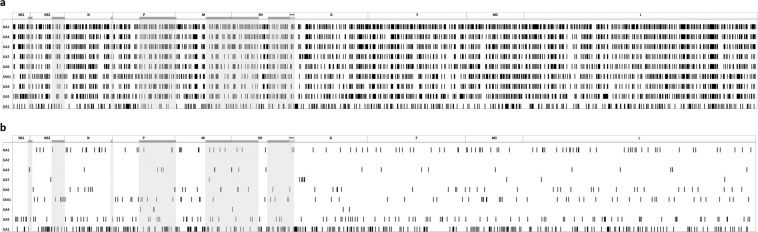
Distribution of molecular markers present in RSV-A genotypes. The location of all (**a**) and unique (**b**) molecular markers present in each genotype is shown.

One thousand two hundered and ninety-four molecular markers were identified distributed throughout the ten genes, and 180 molecular markers throughout the 10 deduced viral protein sequences. Among the 1,294 nucleotide molecular markers, 640 (49.4%) were present in a single genotype, the remaining markers were shared by two or more genotypes. The sensitivity of the 640 unique nucleotide molecular markers to identify a genotype ranged from 75% to 100% (median 98.0%), with specificity ranging from 44% to 100% (median 99.8%). These markers were used to create a map showing characteristic unique patterns for each genotype (Fig. 7). GA1 had 270 nucleotide molecular markers in CDS region and 55 in UTR regions, GA5 91 in CDS and 14 in UTR, NA1 60 in CDS and 9 in UTR, SAA1 61 in CDS and 3 in UTR, GA6 40 in CDS and 7 in UTR, GA3 8 in CDs and 9 in UTR, GA7 8 in CDS, and GA4 2 in CDS and 3 in UTR.

Of the 180 amino acid molecular markers, 76 (42.2%) were present in only one genotype (Supplementary Table 1). The specificity of these markers to identify a genotype ranged from 91 to 100%; of note, the majority of these (75%) were 100% specific. From all amino acid molecular markers, 29 (38.2%) were located in the G protein, 16 (21%) in the L protein, 10 (13.2%) in the F protein, 8 (10.5%) in M2-2, 6 (7.9%) in M2-1, 2 (2.6%) in SH; the remaining proteins had only one molecular marker.

GA1 genotype had 32 unique amino acid molecular markers, GA5 had 16, SAA1 had 9, NA1 had 7, and GA6 had 6, while there were three molecular markers in GA3 and GA4, and there were two molecular markers in GA7. The only genotype without any unique molecular marker (nucleotide or amino acid) was GA2.

### Phylodynamic distribution

Isolation dates and locations were registered for each analyzed sequence (N = 4,414); 12 (0.27%) sequences did not have date and location data, 8 (0.18%) sequences from the United States lacked isolation date information, and 61 (1.38%) sequences were not assigned to a specific genotype since they did not cluster within defined genotype clades.

**Figure 8:**
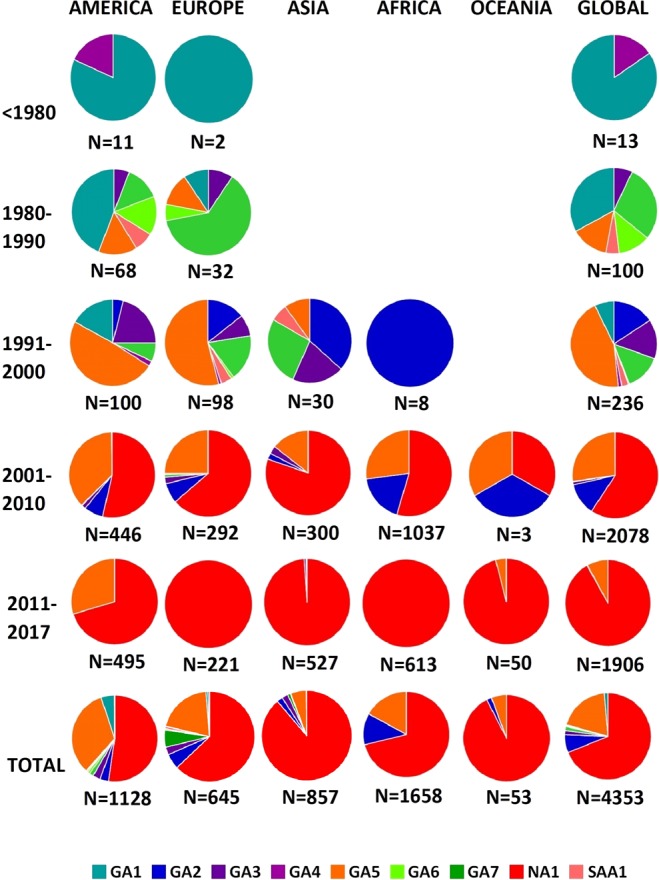
RSV-A genotype distribution before 1980 and each decade thereafter, according to continent of viral detection.

Global circulation patterns of RSV-A genotypes showed at least three important shifting periods in the dominant genotypes (Fig. 8). Initially, between 1957 and 1979, RSV GA1 was the predominating genotype. During a second period (1980–2010) RSV-A showed a wide genomic diversification with a global expansion of multiple genotypes. Finally, a third period (2011 until the present day) was characterized by the worldwide predominance of a single RSV-A genotype; during this period first NA1 genotype and then ON1 lineage were fixed in the population with the extinction of the other genotypes.

## Discussion

Since the identification of RSV, a wide diversity of viral strains has been identified leading to classification into two major groups with multiple genotypes within each of them. However, there is no complete consensus regarding the criteria that define a specific genotype. Genotype classification and assignment is of importance in order to understand the evolution, epidemiology, and clinical presentation of this virus, and has implications regarding the development of vaccines and other preventive interventions. The recent emergence and rapid global extension of new lineages of this virus, such as ON1, highlights the relevance of this issue^14^.

Current classification of RSV-A genotypes is based predominantly on intragenotype and intergentoype p-distance analysis^7,8^. However, cutoff values to define if a viral cluster constitutes a different genotype will vary depending on several factors, such as the length and location of the sequences used for analysis, as well as the number of sequences included in the analysis^12^. For example, analysis of different genes will result in different values, as shown in this study, and may not be sufficiently sensitive to distinguish between genotypes. In addition, the time span of circulation of a genotype will also be reflected in the intragenotypic p-distance; in this regard, it is expected that the intragenotypic p-distance will increase with time and, as diversity within viruses of more recent emergence (such as ON1) accrues, new genotypes are likely to be defined. On the other hand, analyses based on limited regions of the genome, such as the second hypervariable region of the G gene, may result in the identification of subclades within a genotype as separate genotypes. In accordance with some previous reports, we suggest the use of at complete ectodomain of the G gene in order to achieve adequate sensitivity and specificity in genotype identification and assignment^11,12^. In addition, it is important to include a sufficient number of sequences of all existing genotypes in order to obtain consistent results. As an example, current recommendations for identification of new measles virus genotypes indicate that all available sequences of the full coding region of the hemagglutinin gene and of the 450 nucleotides encoding for the carboxyl-terminal region of the nucleoprotein be used for phylogenetic analysis, instead of using only reference sequences^21^. Applying this approach, our analysis corroborated that the GA1, GA4, GA5, GA6, GA7, and SAA1 genotypes are distinct genotypes. As for sequences within the GA2, GA3, and the most recently described genotypes (NA1-NA4 and ON1), we found that these can be grouped into a more limited number of genotypes. Similar results have been reported previously^11^. However, some inconsistencies were encountered when analyzing sequences identified as belonging to the GA2 genotype. One example of this is the assignment of NA4 genotype sequences. In both studies, this genotype was identified to show similarity to GA3 and some GA2 sequences; however, while Trento *et al*. concluded that NA4 should be considered as a GA2 genotype, we conclude that these sequences are better classified within the GA3 genotype, together with a cluster of some sequences previously identified as GA2^11^. Analysis of complete sequences of NS1, NS2, N, SH, F, M2 genes corroborated that NA4 sequences are distinct from GA2 and NA1 genotypes. The other major difference is that we conclude that GA2 and NA1 genotypes are distinct genotypes; the latter included NA1, NA3, ON1, and a clade of sequences previously included as part of the GA2 genotype. Analysis of complete sequences of the NS2, N, M, SH, F, and M2 genes also identified NA1 as a distinct genotype from GA2 sequences. Furthermore, both NA1 sequences and GA3 sequences (the latter including NA4 strains) showed distinct molecular markers in comparison to all other genotypes, including the GA2 genotype. As a result, RSV-A sequences can clearly be divided into nine distinct genotypes: GA1-GA7, SAA1, and NA1. The list of reference sequences used in the present study with genotype reassignment that can be used for future studies is presented in Supplementary Table 2.

Results of phylogenetic analyses of genes other than the G gene were similar to those obtained from analysis of the G gene ectodomain. With some exceptions, the p-distance matrices of all analyzed genes further supported the genotype assignment. Previous studies have also shown that analysis of other genes, such as SH and F, results in clustering of sequences comparable to results obtained from G gene analysis^22,23^. However, due to the lower variability in these genes, separation of sequences belonging to different genotypes may not always be feasible, particularly if complete gene sequences are not used. As an example, G gene and F gene sequences of ON1 viruses identified in Ontario showed similar clustering, but F gene analysis provided lower resolution to differentiate these viruses from closely related strains than the G gene^14^. While genotype classification has usually been based on G gene analysis, since it contains the genomic region with largest variability and, at least for RSV-A, displays the highest evolutionary rate, all RSV genes present evolutionary changes, albeit at a lower rate^10,13,15^. The most notable exceptions regarding the ability of genes other than the G gene to distinguish between genotypes were genoytpes GA4 and SAA1, GA6 and GA7, and GA4 and GA6 for which the p-distance values between them were lower than the intragenotype threshold in several genes. These results were more notable in conserved genes, such as the L gene. We also observed that the number of available sequences had a significant effect on the ability to accurately distinguish between some genotypes, particularly those that have circulated for shorter periods of time. Therefore, distinction between closely related genotypes in genes with low evolutionary rates might be difficult among closely related viruses with a short circulation span. Nevertheless, the position of genotype clades was maintained in the cladograms for all genes, and distinct markers for each genotype can be identified in most genes. As such, analysis of other genes, in addition to the G gene, provides support to the identification and definition of new genotypes.

The most notable recent evolutive change in RSV-A is the emergence and worldwide distribution of viruses with a partial duplication of the G gene^14,24^. Since their identification, these viruses have been considered as a distinct genotype^14^. The original description of these strains as a novel genotype was supported by a p-distance of 0.04 for the ON1 cluster^14^. However, this analysis was based on a fragment of only 264 nucleotides that includes the second hypervariable region of the G gene and included 23 reference strains in addition to the study sequences. Analysis of a larger nucleotidic sequence that includes the complete ectodomain region of the G protein, as suggested by other authors^11,12^, resulted in a high similarity between ON1 and NA1 sequences (p-distance = 0.029). Thus, although ON1 strains show distinct genetic features, including the characteristic 72-nucleotide duplication, the phylogenetic analysis indicates that they do not constitute a separate genotype. In addition, analysis of other genes supported the designation of ON1 sequences within the NA1 genotype. An issue that has been open to question is whether the presence of the partial duplication in ON1 strains by itself should be considered indicative of a new genotype designation^12,25^. We have previously proposed that the emergence of strains with a partial duplication is a dynamic process that resulted from several duplication events^26,27^. In addition, under some circumstances, it is possible that the 72-nucleotide duplication may be lost by ON1 strains^28^. These observations support our proposal that genotype designation should be based on a systematic phylogenetic analysis, independently of the presence of the partial duplication insertion.

In our analysis we used the largest data set of RSV-A sequences that has been reported up until now, since all strains that include at least the G gene ectodomain sequence that were available in GenBank in September 2018 were included (n = 4,414). This allowed us to assess if previously described genotypes can be, in fact, considered as such. As a result, several amino acids that have been reported to be characteristic of some genotypes were found to be present in more than one genotype.

It has previously been reported that amino acids S214, A225, L238, P241, N250, S251, L256, T275, A274, I295, and D297 from the G protein and N125 in the F protein were characteristic of GA5 genotype^9,29–32^. In our study, N250, L238, T275, A274, I295, and D297 were no longer characteristic amino acids of GA5. For example, N250 can also be frequently detected in SAA1 and GA4. Another characteristic previously reported as potentially associated to GA5 is the presence of an amino acid at the 298 position (usually Q298) in the G protein instead of a stop codon^33,34^. However, we found that only 76.8% of GA5 sequences have Q298 and 1.4% of them have a stop codon at position 298.

Previous studies have reported A122, Q156, L215, L226, Y266, T269, S289, L290, and K297 in the G protein as characteristic amino acids of the GA2 genotype^9,30,31^. As a result of definition of NA1 and GA2 as distinct genotypes, none of these amino acids remained as characteristic of GA2, since they can be found in more than one genotype. For example, T269 is a characteristic shared by NA1, GA2, and GA3 genotypes. The presence of this substitution in NA1 genotype has been identified previously^9^. Conversely, with inclusion of NA3, ON1, and some GA2 sequences within the NA1 genotype, some amino acids that have been considered as characteristic of this genotype, such as P215, P230, T264, V279, Y285, and R297 in the G protein, were no longer identified as such^14^.

Since the first description of RSV-A ON1 strains, this virus has been considered as a new genotype^14^. In addition to the partial duplication of the G gene, amino acids L142, G232, N237, K253, Y273, and L314 in the G protein have been reported as characteristic or unique for this genotype^14,26,35–41^. In 2015, Trento *et al*. proposed that ON1, together with NA1, NA2, and NA4 be considered part of the GA2 genotype^11^. Our results confirm that NA1, NA3, and ON1 can be considered as a single genotype. In contrast, we found that GA2 and NA4 form separate clusters from that genotype.

While analysis of the G gene ectodomain allows for the most detailed classification of RSV-A genotypes, we found unique markers for different genoytpes throughout the viral genome. These unique markers could provide a presumptive genotype classification for viruses for which sequence data is available only for genes other than the G gene. Although precise classification using this approach may not be feasible, this could provide some information regarding which RSV genotypes circulated in some regions at specific time periods where information is limited.

In summary, we propose that identification of new genotypes of RSV-A should be based on analysis of, at least, the ectodomain sequence of the G protein gene, and a wide set of reference strains, as well as a large number of sequences of all other genotypes need to be included in the phylogenetic analysis. Intragenotype p-distance for new genotypes should be lower than the lowest intragenotype p-distance of all previously described genotypes (threshold p-distance), and intergenotype p-distance should be higher than the threshold p-distance in comparison to all other previously described genotypes. Phylogenetic analysis of other genes, in addition to the G gene, would provide additional support to the identification of a new genotype.

## Methods

### Selection of sequences and BLAST search

For the present study, we analyzed all RSV sequences available at GenBank that included at least the G gene ectodomain. Sequences were downloaded from NCBI during September 2018. Sequences from synthetic RSV strains, as well as those corresponding to viruses or organisms other than RSV were excluded. In order to identify RSV sequences that fulfilled the inclusion criteria the following search terms were used: (“Respiratory syncytial virus”[Organism] OR Respiratory syncytial virus[All Fields]) NOT attenuated[All Fields] NOT vaccine[All Fields] NOT vaccines[All Fields] NOT chimeric[All Fields] NOT oligomers[All Fields] NOT (“Aspergillus”[Organism] OR aspergillus[All Fields]) NOT (“Rattus”[Organism] OR “Rattus norvegicus”[Organism] OR rat[All Fields]) NOT (“Mus musculus”[Organism] OR mus musculus[All Fields]) NOT (“Bos taurus”[Organism] OR bovine[All Fields]) NOT patent[All Fields] NOT unverified[All Fields] NOT (“unidentified”[Organism] OR unidentified[All Fields]) NOT (“Glycine max”[Organism] OR soybean[All Fields]) NOT (“synthetic construct”[Organism] OR synthetic[All Fields]) NOT (“Klebsiella”[Organism] OR Klebsiella[All Fields]) NOT (“Escherichia”[Organism] OR Escherichia[All Fields]) NOT ribosomal[All Fields] NOT mutant[All Fields] NOT complement[All Fields] NOT acod1[All Fields] AND (“578”[SLEN]: “17000”[SLEN]). In total, 10,340 RSV sequences were obtained.

The resulting 10,340 sequences were then analyzed with the use of BLAST to identify the RSV-A strains. In order to do this, they were divided into local libraries and a local BLAST was carried out using Blast2GO v5.2.5 against a database comprised of 44 RSV-A and 2 RSV-B G gene ectodomain reference sequences^42^; additionally, a cutoff Expect (e) value of E^−10^ was used to discriminate between both viral types. 5,410 sequences of our dataset were categorized as RSV-A by the BLAST search and were once again organized in local libraries and aligned using CLUSTAL Omega algorithm^43^. Then, the G gene sequences were manually inspected, aligned and cropped using BioEdit v7.0.5.3 software^44^. A second cropping was carried out to obtain two different fragments of the G gene for alignment: the first included the complete ectodomain of the G gene (spanning from nt 312 to the end of the G gene) and the second limited to the second hypervariable region (spanning from nt 648 to the end of the G gene)^11^.

All sequences with gaps (other than those resulting from the 72 nt duplication compared to ON1 sequences), degenerate nucleotides, or deletions at the initial, middle, or terminal G ectodomain sequence were excluded from the dataset in order to avoid genotype misassignment. This resulted in a dataset of 4,414 complete G ectodomain sequences; for all these sequences the accession number, strain, year of isolation, country and genotype (assigned by cladistics methods) were recorded. This dataset included 1103 complete genome sequences in which each of the 10 RSV genes (NS1, NS2, N, P, M, SH, G, F, M2, and L) were trimmed from 5′UTR to 3′UTR and aligned using MUSCLE algorithm^43^. The ElimDupes tool (https://www.hiv.lanl.gov/content/ sequence/elimdupesv2/elimdupes.html) was used to remove duplicated sequences on our dataset to perform the phylogenetic analysis.

### Reference sequences selection

An extensive search of genotype reference sequences previously used in the literature was carried out. We defined reference strains as those which have been used in previous studies as reference sequences^11,12,14,30,35,38,45^; among those which had been used as references by several authors, we excluded those in which the genotype assignment was not concordant in all articles. Using these criteria we selected 72 reference sequences that included the genotypes GA1-GA7, NA1-NA4, SAA1, and ON1 genotypes (Supplementary Table 3). In addition, the Long strain sequence was included as an RSV-A prototype^46,47^. Forty-four out of these 73 sequences were equal to or larger than the G gene ectodomain, while 29 only contained the second hypervariable region of the G gene.

Due to the restricted size of 29 out of the 73 reference sequences, equivalent reference sequences were selected to circumvent the lack of the complete ectodomain region sequence of these 29 sequences. Using MEGA X v10.0.3 software, a Maximum Likelihood analysis of the G gene second hypervariable region under the General Time Reversal model with Γ distribution rate (GTR + Γ) and 1,000 bootstrap iterations was performed^48^. Once the Maximum Likelihood analysis had been performed, an equivalent reference sequence was selected for each of the 29 reference sequences that did not contain the complete ectodomain based on the following criteria: adjacent position at the cladogram, bootstrapping value above 90%, and identity percentage above 99%. The equivalent reference sequences are listed in the Supplementary Table 4.

### Genotype assignment

The genotype assignment was performed by clade clustering with the references or equivalent reference sequences via Maximum Likelihood analysis under the GTR + Γ + I model with 1,000 bootstrapping iterations with MEGA X v10.0.3. The genotype assignment was subsequently corroborated with the Maximum Clade Credibility Tree topology generated from the Bayesian Skyline Plot Analysis. To analyze if the identified clusters belonged to distinct genotypes the similarity within and between genotypes, p-distance matrices were generated using MEGA X v10.0.3. Intragenotypic and intergenotypic p-distances were calculated for the G gene ectodomain.

To elucidate the RSV-A phylodynamic changes over time and to corroborate the genotype assignment, a Maximum Clade Credibility Tree was generated from G gene ectodomain unique sequences using Tree Annotator v2.5.1, and the corresponding phylogenetic analysis by the Markov Chain Monte Carlo (MCMC) method was performed with BEAST v2.5.1 package under the (GTR + Γ + I) model^49^. The dataset was analyzed using the Bayesian Skyline method, assuming both a strict and a relaxed molecular clock. MCMC were run 400,000,000 steps and sampled every 20,000 steps; convergence achievement was confirmed with Tracer v1.7.1.

In addition, phylogenetic analysis and p-distance matrices (Including intragenotypic and intergenotypic p-distances) were generated using MEGA X v10.0.3 for the NS1, NS2, N, P, M, SH, F, M2, and L genes.

### Identification of molecular markers

To identify the presence of both nucleotide and amino acid molecular markers between the RSV-A genotypes, complete gene sequences (spanning from 3′UTR to 5′UTR for each gene) from 1,103 RSV strains with complete genome were analyzed. The sequences were grouped according to their previously assigned genotype and the differences of each group with respect to the prototype sequence ATCC VR-26 were recorded. A molecular marker was considered to be present at a site if a nucleotide or amino acid shift occurred with respect to the prototype sequence in 75% or more of the sequences of a given genotype.

## Supplementary information

Supplementary information 

## Data Availability

This study was carried out with data retrieved from GenBank. All data used is available in public databases.
