# Supplementary material for: Respiratory syncytial virus A genotype classification based on systematic intergenotypic and intragenotypic sequence analysis
Source: Sci Rep. 2019 Dec 27;9:20097. doi: 10.1038/s41598-019-56552-2 (PMC6934736; doi:10.1038/s41598-019-56552-2)
Supplement: Supplementary file 1 — Supplementary information [file 41598_2019_56552_MOESM1_ESM.pdf]

Respiratory syncytial virus A genotype classification based on systematic intergenotypic and intragenotypic sequence analysis

Juan Carlos Muñoz-Escalante, Andreu Comas-García, Sofía Bernal-Silva,
Carla Daniela Robles-Espinoza, Guillermo Gómez-Leal, Daniel E. Noyola

Supplementary Figure 1. Distribution of unique and duplicated RSV-A sequences that contain at least the ectodomain of the G gene. In some of the 1,103 viral strains with complete genome sequences, some genes had incomplete sequences, gaps, or degenerate nucleotides. These sequences were excluded from analysis. Therefore, the number of sequences for each gene (unique + duplicated) is less than 1,103.

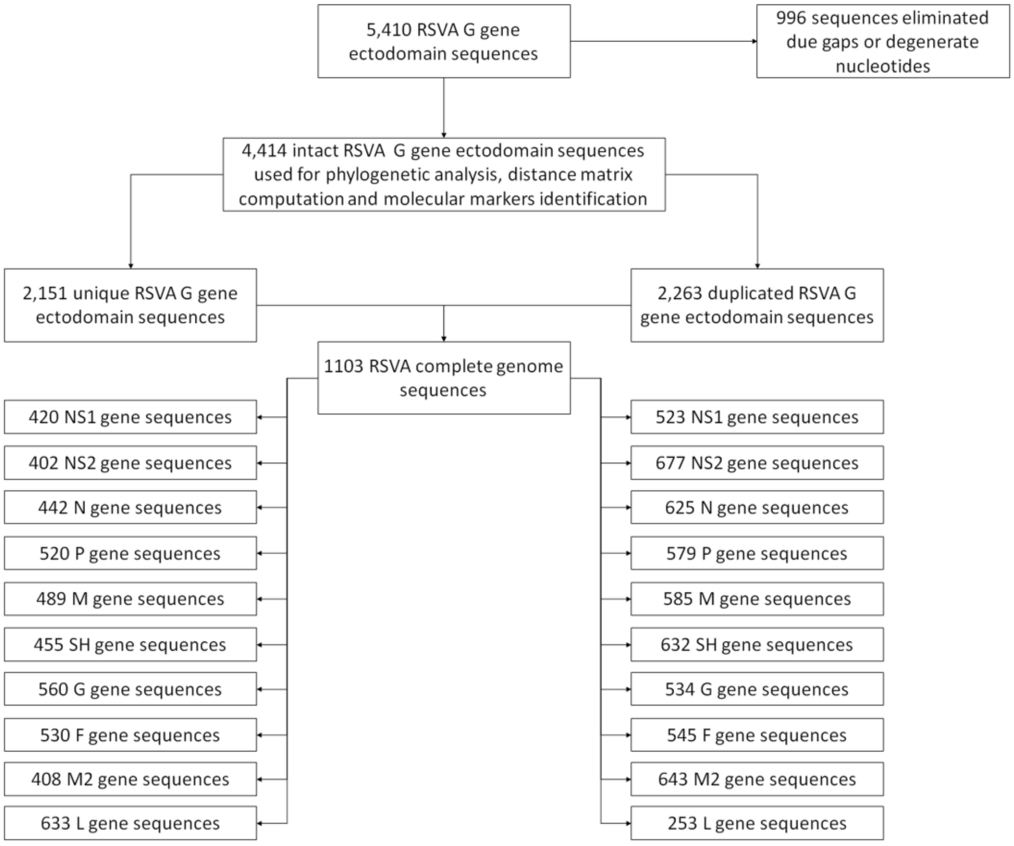

Supplementary Table 1. RSV-A genotype specific amino acids.

Protein	Amino acid substitution	GA1	GA5	GA4	SAA1	GA6	GA7	GA3	GA2	NA1	Sensitivity	Specificity
NS1	I105L	84.5%*									85%	100%
NS2	D7G		87%								87%	100%
N	Y216H	98%									98%	98%
P	E221K				100%						100%	100%
M	C91S	84%									84%	100%
SH	V49I							80%			80%	100%
	L57I				100%						100%	100%
G	F101P	100%									100%	100%
	S102F		98.4%								98%	98%
	L104P	100%									100%	94%
	T107I	100%									100%	100%
	T108I		85.1%								85%	100%
	I114T						100%				100%	99%
	L115P						100%				100%	92%
	N157S	100%									100%	100%
	N161D		89.5%								90%	100%
	N191S		99.1%								99%	100%
	K197R	75.0%									75%	100%
	T210I				91.6%						92%	100%
	K221T					92%					92%	91%
	P222S	100%									100%	99%
	V225A		97.8%								98%	95%
	K229N					92.3%					92%	100%
	T238I		93.3%								93%	100%
	T241P		96%								96%	100%
	N242S			100%							100%	95%
	L247M			100%							100%	100%
	T249I			100%							100%	99%
	N251S		98.5%								99%	100%
	G254R	81%									81%	99%
	P256L		82.7%								83%	99%
	V279I		90.3%								90%	100%
	Y280S	100%									100%	100%
	P/L314S	98.6%									99%	100%
	P316S									99.9%	100%	100%
	T320P	86.9%									87%	99%
F	L6I	75.9%									76%	100%
	K20F	81%									81%	100%
	N105S									94.1%	94%	100%
	Y122A	96.2%									96%	100%

	N124K	93.2%									93%	100%
	T125N		87.3%								87%	99%
	E356D	75.9%									76%	99%
	I384V							100%			100%	100%
	S466N							100%			100%	100%
	L547F		99.6%								100%	96%
M2-1	P120L	99.1%									99%	100%
	I132T				100%						100%	98%
	S179L	84.6%									85%	100%
	T180A				100%						100%	100%
	V181D				100%						100%	100%
	S182N		98.4%								95%	99%
M2-2	S48P									99%	99%	100%
	N16T	99.1%									91%	100%
	N23R	99.1%									99%	100%
	Q42P	99.1%									99%	100%
	P52T	99.1%									99%	100%
	A64I	99.1%									99%	100%
	N69S	78%									78%	100%
	D78E	99.1%									99%	100%
L	I103T									92.1%	92%	100%
	D174G		99.6%								99%	100%
	H177Q									98.3%	98%	100%
	I232T	100%									100%	100%
	H255N	86%									86%	100%
	N374D				100%						100%	100%
	V388I									97%	97%	100%
	V455I	100%									100%	100%
	E575D									97%	97%	100%
	M646L					90%					90%	99%
	V821I	100%									100%	100%
	K960N				100%						100%	100%
	H1690Y					90%					90%	100%
	N1724D		99.6%								99%	100%
	L1729Q				100%						100%	100%
	D1731E	86.0%									86%	100%

*Proportion of sequences that present the specific amino acid change in the genotype in which the molecular marker is considered as specific

Supplementary Table 2. Revised RSV-A reference sequences

Number	Accession	Genotype		Number	Accession	Genotype
1	M11486.1	GA1		38	KU316133.1	GA5
2	Z33427.1	GA1		39	AY343586.1	GA5
3	Z33431.1	GA1		40	JX198135.1	GA5
4	Z33432.1	GA1		41	AF193318.1	GA5
5	AF065257.1	GA1		42	HQ731701.1	GA5
6	AF065407.1	GA1		43	KJ627695.1	GA5
7	AF233914.1	GA1		44	JX513382.1	GA5
8	JF920069.1	GA1		45	AY343578.1	GA5
9	KU316164.1	GA1		46	JX015487.1	GA5
10	KU316099.1	GA1		47	KP258723.1	GA6
11	M17212.1	GA1		48	DQ985131.1	GA6
12	AY114149.1	GA2		49	Z33455.1	GA7
13	HQ731710.1	GA2		50	Z33417.1	GA7
14	JQ901453.1	GA2		51	AF348804.1	GA7
15	Z33422.1	GA3		52	AF193320.1	GA7
16	AF065256.1	GA3		53	HQ731703.1	GA7
17	Z33424.1	GA3		54	X73351.1	GA7
18	MG642050.1	GA3		55	KP792358.1	NA1
19	AY343615.1	GA3		56	KP792359.1	NA1
20	DQ985123.1	GA3		57	KF300972.1	NA1
21	DQ985122.1	GA3		58	JX015495.1	NA1
22	Z33416.1	GA3		59	KC297292.1	NA1
23	Z33426.1	GA3		60	KC297277.1	NA1
24	Z33414.1	GA3		61	KC297260.1	NA1
25	AF233905.1	GA3		62	KF300973.1	NA1
26	AF193317.1	GA3		63	JN257694.1	NA1
27	AF193319.1	GA3		64	KP792361.1	NA1
28	AY343621.1	GA3		65	KP792365.1	NA1
29	KC297381.1	GA3		66	KP792362.1	NA1

30	KC297324.1	GA3		67	KP792374.1	NA1
31	KC297374.1	GA3		68	KP792370.1	NA1
32	AF065254.1	GA4		69	KP792373.1	NA1
33	AY114151.1	GA5		70	KP792375.1	NA1
34	Z33494.1	GA5		71	AY911262.1	PRO
35	Z33430.1	GA5		72	KP258696.1	SAA1
36	AF065255.1	GA5		73	DQ985132.1	SAA1
37	AY114150.1	GA5				

Supplementary Table 3. RSV-A reference sequences

Number	Accession	Year	Country	Continent	Genotype	Genome	Equivalent	Reference	Source
1	M11486.1	1985	United States	America	GA1	C.	-	1	2
2	Z33427.1	1990	Uruguay	America	GA1	C. cds	-	3	2
3	Z33431.1	1991	Uruguay	America	GA1	C. cds	-	3	4, 5,2
4	Z33432.1	1992	Uruguay	America	GA1	C. cds	-	3	4, 2
5	AF065257.1	1998	United States	America	GA1	C. cds	-	6	2
6	AF065407.1	1998	United States	America	GA1	C. cds	-	7	2
7	AF233914.1	2000	United States	America	GA1	HVR	-	8	2
8	AF233902.1	2000	United States	America	GA1	HVR	KU316164.1	8	2
9	AF233917.1	2000	United States	America	GA1	HVR	KU316099.1	8	5, 2
10	JF920069.1	2007	United States	America	GA1	C.	-	9	10
11	Z33422.1	1989	Uruguay	America	GA2	C. cds	-	3	2
12	AF448498.1	1994	Uruguay	America	GA2	HVR	DQ985122.1	11	5, 2
13	AF065256.1	1998	United States	America	GA2	C. cds	-	6	2
14	AF233900.1	2000	United States	America	GA2	HVR	MG642050.1	8	2
15	AF233915.1	2000	United States	America	GA2	HVR	Z33424.1	8	2
16	AF233923.1	2000	United States	America	GA2	HVR	AY343615.1	8	5, 2
17	AY114149.1	2002	Singapore	Asia	GA2	P.	-	12	2
18	AY114151.1	2002	Singapore	Asia	GA2	P.	-	12	5, 2
19	AY146435.1	2002	South Africa	Africa	GA2	HVR	HQ731710.1	13	5, 2
20	AY472086.1	2003	Brazil	America	GA2	HVR	DQ985123.1	14	2
21	Z33416.1	1990	Spain	Europe	GA3	C. cds	-	3	5, 15
22	Z33426.1	1990	Uruguay	America	GA3	C. cds	-	3	5, 2
23	Z33414.1	1993	Spain	Europe	GA3	C. cds	-	3	5, 15
24	AF233905.1	2000	Canada	America	GA3	HVR	-	8	5, 2
25	AF233913.1	2000	United States	America	GA3	HVR	AF193317.1	8	5, 2
26	AF233920.1	2000	United States	America	GA3	HVR	AY343621.1	8	2
27	AF233921.1	2000	United States	America	GA3	HVR	AF193319.1	8	5, 2
28	AF065254.1	1998	ND	ND	GA4	C. cds	-	6	2
29	Z33494.1	1990	Uruguay	America	GA5	C. cds	-	3	5, 2
30	Z33430.1	1992	Uruguay	America	GA5	C. cds	-	3	5, 2
31	AF065255.1	1998	ND	ND	GA5	C. cds	-	8	5, 2
32	AF233906.1	2000	Canada	America	GA5	HVR	HQ731701.1	8	2
33	AF233903.1	2000	United States	America	GA5	HVR	AF193318.1	8	2
34	AF233909.1	2000	United States	America	GA5	HVR	AY343586.1	8	2
35	AF233916.1	2000	United States	America	GA5	HVR	KJ627695.1	8	2
36	AF233919.1	2000	United States	America	GA5	HVR	JX198135.1	8	2
37	AF348803.1	2001	South Africa	Africa	GA5	HVR	KU316133.1	16	2
38	AY146437.1	2002	South Africa	Africa	GA5	HVR	JX015487.1	13	5, 2
39	AY114150.1	2003	Singapore	Asia	GA5	P.	-	12	2
40	AY472094.1	2003	South Africa	Africa	GA5	HVR	JX513382.1	14	2
41	AB175815.1	2004	Japan	Asia	GA5	HVR	AY343578.1	17	2
42	AF233901.1	2000	United States	America	GA6	HVR	DQ985131.1	8	2
43	AF233918.1	2000	United States	America	GA6	HVR	KP258723.1	8	2
44	Z33455.1	1992	Spain	Europe	GA7	C. cds	-	3	2
45	Z33417.1	1992	Spain	Europe	GA7	C. cds	-	3	2
46	AF233904.1	2000	Canada	America	GA7	HVR	HQ731703.1	8	2
47	AF233907.1	2000	Canada	America	GA7	HVR	AF193320.1	8	2
48	AF233910.1	2000	United States	America	GA7	HVR	X73351.1	8	2
49	AF348804.1	2001	South Africa	Africa	GA7	HVR	-	16	2
50	AB470478.1	2004	Japan	Asia	NA1	HVR	JX015495.1	18	5
51	KP792358.1	2007	Spain	Europe	NA1	C. cds	-	15	15
52	KP792359.1	2008	Spain	Europe	NA1	C. cds	-	15	15
53	KF300972.1	2010	Panama	America	NA1	C. cds	-	19	15

54	JX256960.1	2003	Malaysia	Asia	NA2	HVR	JQ901453.1	20	21
55	KC297292.1	2011	China	Asia	NA3	C. cds	-	22	22
56	KC297277.1	2011	China	Asia	NA3	C. cds	-	22	22
57	KC297260.1	2011	China	Asia	NA3	C. cds	-	22	22
58	KC297381.1	2007	China	Asia	NA4	C. cds	-	22	22
59	KC297324.1	2011	China	Asia	NA4	C. cds	-	22	22
60	KF300973.1	2010	Panama	America	ON1	C. cds	-	19	15
61	JN257694.1	2011	Canada	America	ON1	C. cds	-	5	5, 15
62	KP792361.1	2012	Spain	Europe	ON1	C. cds	-	15	15
63	KP792365.1	2012	Spain	Europe	ON1	C. cds	-	15	15
64	KP792362.1	2012	Spain	Europe	ON1	C. cds	-	15	15
65	KP792374.1	2012	Spain	Europe	ON1	C. cds	-	15	15
66	KP792370.1	2012	Spain	Europe	ON1	C. cds	-	15	15
67	KP792373.1	2013	Spain	Europe	ON1	C. cds	-	15	15
68	KP792375.1	2014	Spain	Europe	ON1	C. cds	-	15	15
69	M17212.1	1987	United States	America	PRO	C. cds	-	23	2, 15
70	AY911262.1	2005	United States	America	PRO	C.	-	24	24
71	AF348807.1	2001	South Africa	Africa	SAA1	HVR	DQ985132.1	16	2, 15
72	AF348808.1	2001	South Africa	Africa	SAA1	HVR	KP258696.1	16	2, 15

1. Wertz, G. W. *et al.* Nucleotide sequence of the G protein gene of human respiratory syncytial virus reveals an unusual type of viral membrane protein. *Proc Natl Acad Sci U S A* **82**, 4075–4079 (1985).
2. Reiche, J. & Schweiger, B. Genetic variability of group A human respiratory syncytial virus strains circulating in Germany from 1998 to 2007. *J. Clin. Microbiol.* **47**, 1800–1810 (2009).
3. García, O. *et al.* Evolutionary pattern of human respiratory syncytial virus (subgroup A): cocirculating lineages and correlation of genetic and antigenic changes in the G glycoprotein. *J. Virol.* **68**, 5448–59 (1994).
4. Agoti, C. N., Otieno, J. R., Gitahi, C. W., Cane, P. A. & Nokes, D. J. Rapid spread and diversification of respiratory syncytial virus genotype ON1, Kenya. *Emerg. Infect. Dis.* **20**, 950–959 (2014).
5. Eshaghi, A. R. *et al.* Genetic variability of human respiratory syncytial virus a strains circulating in Ontario: A novel genotype with a 72 nucleotide G gene duplication. *PLoS One* **7**, (2012).
6. Peret, T., Golub, J., Anderson, L., Hall, C. & Schnabel, K. Circulation patterns of genetically distinct group A and B strains of human respiratory syncytial virus in a community. *J. Gen. Virol.* **79**, 2221–2229 (1998).
7. Sullender, W. M., Mufson, M. A., Prince, G. A., Anderson, L. J. & Wertz, G. W. Antigenic and genetic diversity among the attachment proteins of group A respiratory syncytial viruses that have caused repeat infections in children. *J. Infect. Dis.* **178**, 925–932 (1998).
8. Peret, T. C. T. *et al.* Circulation Patterns of Group A and B Human Respiratory Syncytial Virus Genotypes in 5 Communities in North America. *J. Infect. Dis.* **181**, 1891–1896 (2000).
9. Rebuffo-Scheer, C. *et al.* whole genome sequencing and evolutionary analysis of human respiratory syncytial virus A and B from Milwaukee , WI 1998-2010. *PLoS One* **6**, e25468 (2011).
10. Agoti, C. *et al.* Successive respiratory syncytial virus epidemics in local populations

arise from multiple variant introductions, providing insights into virus persistence. *J. Virol.* **89**, 11630–11642 (2015).
11. Frabasile, S. *et al.* Antigenic and genetic variability of human respiratory syncytial viruses (group A) isolated in Uruguay and Argentina : 1993 – 2001. *J. Med. Virol.* **71**, 305–312 (2003).
12. Lim, C. S., Kumarasinghe, G. & Chow, V. T. K. Sequence and phylogenetic analysis of SH, G, and F genes and proteins of human respiratory syncytial virus isolates from Singapore. *Acta Virol.* **47**, 97–104 (2003).
13. Madhi, S. A. *et al.* Respiratory syncytial v irus associated illness in high-risk children and national characterisation of the circulating virus genotype in South Africa. *J. Clin. Virol.* **27**, 180–189 (2013).
14. Moura, F. E. *et al.* Genetic diversity of respiratory syncytial virus isolated during an epidemic period from children of Northeastern Brazil. *J. Med. Virol.* **74**, 156–160 (2004).
15. Trento, A. *et al.* Conservation of G-protein epitopes in respiratory syncytial virus (Group A) despite broad genetic diversity: Is antibody selection involved in virus evolution? *J. Virol.* **89**, 7776–7785 (2015).
16. Venter, M., Madhi, S. A., Tiemessen, C. T. & Schoub, B. D. Genetic diversity and molecular epidemiology of respiratory syncytial virus over four consecutive seasons in South Africa: identification of new subgroup A and B genotypes. *J. Gen. Virol.* **82**, 2117–2124 (2001).
17. Sato, M. *et al.* Molecular epidemiology of respiratory syncytial virus infections among children with acute respiratory symptoms in a community over three seasons. *J. Clin. Microbiol.* **43**, 36–40 (2005).
18. Shobugawa, Y. *et al.* Emerging genotypes of human respiratory syncytial virus subgroup A among patients in Japan. *J. Clin. Microbiol.* **47**, 2475–2482 (2009).
19. Abrego, L. *et al.* Genetic characterization of human respiratory syncytial virus isolated in Panama 2008-2012. *Unpublished* doi:10.4049/jimmunol.1003584
20. Khor, C. S., Sam, I. C., Hooi, P. S. & Chan, Y. F. Displacement of predominant respiratory syncytial virus genotypes in Malaysia between 1989 and 2011. *Infect. Genet. Evol.* **14**, 357–360 (2013).
21. Pierangeli, A. *et al.* Rapid spread of the novel respiratory syncytial virus A ON1 genotype, central Italy, 2011 to 2013. *Euro Surveill.* **19**, 1–15 (2014).
22. Cui, G. *et al.* Rapid replacement of prevailing genotype of human respiratory syncytial virus by genotype ON1 in Beijing, 2012-2014. *Infect. Genet. Evol.* **33**, 163–168 (2015).
23. Johnson, P. R., Spriggs, M. K., Olmsted, R. A. & Collins, P. L. The G glycoprotein of human respiratory syncytial viruses of subgroups A and B: extensive sequence divergence between antigenically related proteins. *Proc Natl Acad Sci U S A* **84**, 5625–5629 (1987).
24. Lo, M. S., Brazas, R. M. & Holtzman, M. J. Respiratory syncytial virus nonstructural proteins NS1 and NS2 mediate inhibition of Stat2 expression and alpha/beta interferon responsiveness. *J. Virol.* **79**, 9315–9319 (2005).

Supplementary Table 4. RSV-A equivalent reference sequences

Number	Accession	Year	Country	Continent	Genotype	Genome	Equivalent
1	KU316164.1	1995	United States	America	GA1	C. genome	AF233902.1
2	KU316099.1	1997	United States	America	GA1	C. genome	AF233917.1
3	Z33424.1	1988	Uruguay	America	GA2	C. cds	AF233915.1
4	MG642050.1	1994	United States	America	GA2	C. genome	AF233900.1
5	AY343615.1	1994	Belgium	Europe	GA2	P. cds	AF233923.1
6	DQ985123.1	1997	Belgium	Europe	GA2	P. cds	AY472086.1
7	HQ731710.1	1997	Great Britain	Europe	GA2	P. cds	AY146435.1
8	DQ985122.1	2005	Belgium	Europe	GA2	P. cds	AF448498.1
9	AF193317.1	1995	Korea	Asia	GA3	P. cds	AF233913.1
10	AF193319.1	1995	Korea	Asia	GA3	P. cds	AF233921.1
11	AY343621.1	1995	Belgium	Europe	GA3	P. cds	AF233920.1
12	KU316133.1	1990	United States	America	GA5	C. genome	AF348803.1
13	AY343586.1	1993	Belgium	Europe	GA5	P. cds	AF233909.1
14	JX198135.1	1994	United States	America	GA5	P. genome	AF233919.1
15	AF193318.1	1995	Korea	Asia	GA5	P. cds	AF233903.1
16	HQ731701.1	1996	Great Britain	Europe	GA5	P. cds	AF233906.1
17	KJ627695.1	2001	United States	America	GA5	C. genome	AF233916.1
18	JX513382.1	2001	Brazil	America	GA5	P. cds	AY472094.1
19	AY343578.1	2001	Belgium	Europe	GA5	P. cds	AB175815.1
20	JX015487.1	2006	Netherlands	Europe	GA5	C. genome	AY146437.1
21	KP258723.1	1986	United States	America	GA6	C. genome	AF233918.1
22	DQ985131.1	1998	Belgium	Europe	GA6	P. cds	AF233901.1
23	AF193320.1	1995	Korea	Asia	GA7	P. cds	AF233907.1
24	HQ731703.1	1996	GreatBritain	Europe	GA7	P. cds	AF233904.1
25	X73351.1	ND	ND	ND	GA7	C. cds	AF233910.1
26	JX015495.1	2008	Netherlands	Europe	NA1	C. genome	AB470478.1
27	JQ901453.1	2002	Netherlands	Europe	NA2	C. genome	JX256960.1
28	KP258696.1	1986	United States	America	SAA1	C. genome	AF348808.1
29	DQ985132.1	1997	Belgium	Europe	SAA1	P. cds	AF348807.1
